# Preventive and therapeutic challenges in combating Zika virus infection: are we getting any closer?

**DOI:** 10.1007/s13365-017-0513-4

**Published:** 2017-01-23

**Authors:** Meera V. Singh, Emily A. Weber, Vir B. Singh, Nicole E. Stirpe, Sanjay B. Maggirwar

**Affiliations:** Department of Microbiology and Immunology, University of Rochester School of Medicine and Dentistry, 601 Elmwood Avenue, Rochester, NY 14642 USA

**Keywords:** Zika virus, Microcephaly, Antibody dependent enhancement of infection, Therapeutics

## Abstract

The neuroteratogenic nature of Zika Virus (ZIKV) infection has converted what would have been a tropical disease into a global threat. Zika is transmitted vertically via infected placental cells especially in the first and second trimesters. In the developing central nervous system (CNS), ZIKV can infect and induce apoptosis of neural progenitor cells subsequently causing microcephaly as well as other neuronal complications in infants. Its ability to infect multiple cell types (placental, dermal, and neural) and increased environmental stability as compared to other flaviviruses (FVs) has broadened the transmission routes for ZIKV infection from vector-mediated to transmitted via body fluids. To further complicate the matters, it is genetically similar (about 40%) with the four serotypes of dengue virus (DENV), so much so that it can almost be called a fifth DENV serotype. This homology poses the risk of causing cross-reactive immune responses and subsequent antibody-dependent enhancement (ADE) of infection in case of secondary infections or for immunized individuals. All of these factors complicate the development of a single preventive vaccine candidate or a pharmacological intervention that will completely eliminate or cure ZIKV infection. We discuss all of these factors in detail in this review and conclude that a combinatorial approach including immunization and treatment might prove to be the winning strategy.

## Background

Multiple reports have now confirmed that Zika virus (ZIKV) is a neuroteratogenic pathogen and is the causative agent of microcephaly and other developmental anomalies of the central nervous system (CNS) in children born to infected mothers (Cugola et al. 2016; Franca et al. 2016; Li et al. 2016a; Miner et al. 2016; Pacheco et al. 2016; Roberts and Frosch 2016). Not only that, but it is also associated with Guillain-Barre syndrome (GBS) (Araujo et al. 2016; Cao-Lormeau et al. 2016; Roze et al. 2016), thrombocytopenia (Karimi et al. 2016; Sharp et al. 2016), and ocular complications (Moshfeghi et al. 2016; Parke et al. 2016; Valentine et al. 2016; Ventura et al. 2016) in infected individuals. It is a mosquito-borne flavivirus (FV) that was isolated in 1947 from a rhesus monkey in the Zika forest of Uganda (Dick 1952; Dick et al. 1952) and has caused major epidemics in Micronesia, French Polynesia, and South and Central America since 2007 (Weaver et al. 2016; White et al. 2016). The severity of the current epidemic has prompted massive research effort to understand and eradicate this global health concern.

Lot of meaningful data has been generated in a very short time covering different aspects of the infection, from its association with microcephaly to mechanistic insights into neuropathogenesis as well as placental transmission; however, we are yet to come up with an efficient strategy to tackle this eminent threat. The purpose of this review is to examine the factors that, we deem, play a very decisive role in designing preventive and therapeutic measures to combat ZIKV infection.

## Environmental stability and cellular tropism of ZIKV

While most of the FVs are mainly transmitted through vector bite (except for hepatitis C virus, which is a non-arboviral FV) (Blitvich and Firth 2015), ZIKV has been shown to be transmitted sexually as well as vertically. It can persist in semen for longer time (Harrower et al. 2016; Mansuy et al. 2016; Matheron et al. 2016; Reusken et al. 2016; Turmel et al. 2016) and was also detected in breast milk (Dupont-Rouzeyrol et al. 2016), saliva, and urine (Bonaldo et al. 2016; Fourcade et al. 2016; Liuzzi et al. 2016; Wiwanitkit 2016). Further, it was found to retain most of its infectivity during a pH range of 5–11 (Muller et al. 2016), is much more stable at higher temperatures as compared to other FVs, and can remain infectious for up to 3 days in the form of dried spots (Kostyuchenko et al. 2016; Muller et al. 2016). Investigators led by Kostuchenko speculated that ZIKV expands into smooth-surfaced particles when incubated in harsher conditions making the lipid envelope more fluid and then allowing it to revert back to its normal state. They attributed this to a one-residue insertion (Ala340) in the DIII domain of E protein, which helps in forming an extra hydrogen bond with other interacting DIII domains of nearby E proteins (Kostyuchenko et al. 2016). These are important factors to be kept in consideration while developing preventive/therapeutic approaches against ZIKV.

Further, ZIKV also has a very broad tropism and is known to use Axl, Tim1, Tyro3, and DC-SIGN as entry receptors in different cell types. ZIKV was shown to infect and replicate in human dermal fibroblasts, epidermal keratinocytes, and immature dendritic cells in vitro (Hamel et al. 2015). Miner and colleagues found virus in the placental trophoblasts of mice infected with Zika (Miner et al. 2016). Additionally, Zika virus has been found to replicate in several human trophoblast cell lines, but not trophoblast cells from full term placentas that release type II IFN (Bayer et al. 2016; El Costa et al. 2016; Miner et al. 2016). Tabata et al. infected different primary cell types from mid- and late-gestation placentas and explants from first-trimester chorionic villi. They found that ZIKV-infected cytotrophoblasts, endothelial cells, fibroblasts, and Hofbauer cells in chorionic villi and amniotic epithelial cells and trophoblast progenitors in amniochorionic membranes. Furthermore, it ﻿infected these cells with higher viral titers in mid gestation versus late gestation (Tabata et al. 2016). Further, multiple studies have indicated that ZIKV infects neural stem cell progenitors, radial glia, as well as astrocytes (Brault et al. 2016; Cugola et al. 2016; Dang et al. 2016; Garcez et al. 2016; Hughes et al. 2016; Li et al. 2016b; Nowakowski et al. 2016; Tang et al. 2016). This data suggests that ZIKV can infect the fetus through placental transmission early in gestation by infecting multiple placental cell types in the first or second trimesters. Once inside the developing fetus, ZIKV targets neural progenitor cells and hinders their differentiation and subsequently causes microcephaly (Cugola et al. 2016; Dang et al. 2016; Garcez et al. 2016; Hughes et al. 2016; Li et al. 2016a; Tang et al. 2016).

## Zika-associated microcephaly and other cortical malformations

FVs can be broadly categorized into two types based on their pathogenic effects as hemorrhagic viruses, such as dengue virus (DENV), yellow fever virus (YFV), and encephalitic viruses, like West Nile virus (WNV), Japanese encephalitis virus (JEV), and now ZIKV. Other than prenatal death (van der Eijk et al. 2016), microcephaly is by far the most severe and irreversible complication associated with ZIKV infection. It results in a cerebral cortex that is reduced in size, whereas overall organization of the brain is mostly unaffected. However, some cases are also known to be associated with abnormal gyral pattern (microlissencephaly, i.e., smooth agyrus brain) and other brain malformations (Gilmore and Walsh 2013; Mlakar et al. 2016; Strafela et al. 2016).

A recent ZIKV outbreak in northeast Brazil was associated with a rise in infants born with microcephaly (Schuler-Faccini et al. 2016). Another study examining the physiology of neonates and fetuses from ZIKV positive pregnant women found incedences of microcephaly, cerebral calcifications, and intrauterine growth restriction (Brasil et al. 2016). In some cases, ZIKV infection was also linked to early fetal death (Brasil et al. 2016; Franca et al. 2016; Meaney-Delman et al. 2016). In yet another report, close examination of 1501 suspected cases of congenital Zika virus syndrome found that 83% of definite or probable cases could be identified by microcephaly (Franca et al. 2016). More recently, data from the epidemiological reports of Health Surveillance (Brazilian Ministry of Health) indicates that of the 1656 confirmed cases of microcephaly or other neurological abnormalities, 15.4% were associated with ZIKV infection (Magalhaes-Barbosa et al. 2016). This causal link between the increase of microcephaly along with other fetal impairments and ZIKV infection in pregnant women has been strengthened by virologic evidence. Researchers in French Polynesia reviewed stored samples of amniotic fluid from cases with fetal cerebral anomaly and microcephaly to discover that four out of five specimens were found positive for ZIKV (Besnard et al. 2016). Similarly, amniotic fluid from two pregnant women in Brazil whose fetuses had microcephaly tested positive for ZIKV (Calvet et al. 2016). Additional studies have found the presence of ZIKV directly in the microcephalic fetal brain tissues (Driggers et al. 2016; Mlakar et al. 2016; Sarno et al. 2016; Schuler-Faccini et al. 2016). Several murine models have provided crucial evidence confirming the relationship between ZIKV and fetal microcephaly. Studies in which pregnant SJL mice were given a ZIKV strain isolated from northeast Brazil resulted in progeny’s brain tissue containing viral RNA and microcephaly-associated malformations, such as reduced cell numbers and cortical layer thickness (Cugola et al. 2016). Similar defects were observed in pups following ZIKV infection of pregnant mice that lacks type I interferon signaling (A129 mice) (Li et al. 2016a; Miner et al. 2016). All of these reports conclusively establish ZIKV as a causative agent for microcephaly.

Due to the lack of systematic documentation or scarcity of longitudinally collected clinical specimen, it remains unclear if ZIKV infection during specific trimester serves as a risk factor. However, a strong association between fetal microcephaly and infection in the first trimester or early in the second trimester has been determined based on data collected from both Brazil and French Polynesia (Cauchemez et al. 2016; Franca et al. 2016; Johansson et al. 2016; Reefhuis et al. 2016; Schuler-Faccini et al. 2016). Additional data recently collected from a study in Colombia suggests that infection in the third trimester is not closely linked with any defects in the fetus (Pacheco et al. 2016). This trend in infection timing and outcome is similar to other pathogens that infect the fetus such as, Rubella, cytomegalovirus (CMV), and *Toxoplasma gondii* (Jenum et al. 1998; Mathur et al. 1982; Miller et al. 1982; Pass et al. 2006). However, there is still a concern that ZIKV could be dangerous for fetuses even in the third trimester. Reports have shown that abnormal fetus outcomes with infections up to 36 weeks of gestation (Brasil et al. 2016; Soares de Souza et al. 2016).

Despite the strong evidence that shows association of fetal neuronal defects with ZIKV infection, the molecular pathways involved in ZIKV-associated microcephaly are largely unknown except for two recent reports. First, an excellent study by Liang et al. showed that ZIKV NS4A and NS4B proteins induce autophagy in human fetal NSCs by deregulating Akt-mTOR signaling (Liang et al. 2016b), whereas Dang et al. showed that ZIKV depletes neural progenitors through activation of Toll-like receptor 3 (TLR3) (Dang et al. 2016). In addition, much can be learned from what is known about microcephaly vera (true microcephaly), an autosomal recessive neurodevelopmental defect. There are multiple genes associated with microcephaly vera, including MCPH1, ASPM, CDK5RAP2, CENPJ, STIL, WDR62, CEP135, CEP152, CEP63, and CEP152, all of which encode for proteins associated with centrosome, mitotic spindles, or centrioles (David et al. 2014; Gilmore and Walsh 2013). During neurogenesis, the fate of dividing neuronal precursors is very critical in defining the ultimate size of the cortex. Nearly all neurons in the cerebral cortex complete proliferation by mid-gestation, and none are generated after birth (Spalding et al. 2005). Further in more complex vertebrate CNS, the newly specified neurons migrate to a specific location before they differentiate and form synapses (Cooper 2013). Hence, it has been proposed that microcephaly is caused by reduced number of neurons owing to defective mitoses of fetal neuronal precursor cells as well as impaired migration of the neurons to designated areas of the brain.

Li and coworkers found decreased expression of most of the above-mentioned genes in microcephalic progeny of IFNRI/II knockout mice infected with ZIKV (Li et al. 2016a). Further, in vitro studies have demonstrated that ZIKV induces cell death in human induced pluripotent stem cell (iPS)-derived neural stem cells and disrupts the formation of neurospheres and reduces the growth of brain organoids (Cugola et al. 2016; Dang et al. 2016; Garcez et al. 2016). Hughes et al. infected various neuroblastoma cell lines at different stages of differentiation with PRVABC strain of ZIKV and showed that only the undifferentiated cells were permissive to infection (Hughes et al. 2016). Brault et al. demonstrated that ZIKV and WNV both showed neurotropism in in vitro infection experiments; however, only ZIKV impaired cell cycle progression of neural stem cells (Brault et al. 2016). All of these findings together indicate that ZIKV infection has a teratogenic window, mostly occurring in the first trimester, while the neuronal stem cells are still differentiating. During this time period, maternal infection can lead to microcephaly by targeting cortical progenitor cells, inducing cell death as well as impaired neuronal migration.

## Other ZIKV-associated health sequelae

Another negative neurological association of ZIKV infection is the occurrence of GBS (Araujo et al. 2016; Cao-Lormeau et al. 2016; Dos Santos et al. 2016; Roze et al. 2016). It is an acute, immune-mediated polyradiculoneuropathy typically occurring after a range of infections including upper respiratory infections, like influenza, and digestive tract infections, notably *Campylobacter jejuni* and CMV or Epstein-Barr virus infections (Araujo et al. 2016; Cao-Lormeau et al. 2016; Roze et al. 2016). It is not clear what causes the onset of GBS; however, different mechanisms including molecular mimicry, epitope spreading, bystander activation, and production of superantigens have been proposed (Anaya et al. 2016). In axonal variants of GBS, the presence of a broad range of anti-glycolipid IgG antibodies directed to gangliosides has been described (Rinaldi and Willison 2008; Willison 2007). Interestingly, Cao-Lormeau and colleagues found that 41 of the 42 individuals diagnosed with GBS during the ZIKV epidemic in 2013–2014 in French Polynesia had anti-ZIKV virus IgM or IgG and had experienced a transient illness in a median of 6 days before the onset of neurological symptoms, suggesting a recent ZIKV infection (Cao-Lormeau et al. 2016). All of these individuals were seronegative for any other infections known to be associated with GBS. However, they found a less than 50% occurrence of the anti-glycolipid antibodies in these individuals suggesting that ZIKV-associated GBS pathogenesis might involve other mechanisms in addition to autoimmune response against glycolipids/gangliosides.

In addition to neurologic complications, ZIKV infection might be associated with hematologic abnormalities as well. So far, there have been three reported cases (in two independent studies) of ZIKV-associated severe thrombocytopenia and subcutaneous hematomas (Karimi et al. 2016; Sharp et al. 2016). Thrombocytopenia is also associated with DENV infection (de Azeredo et al. 2015). Studies have reported increased thrombopoietin (TPO) levels in the blood of DENV-infected individuals (Matondang et al. 2004), and that DENV inhibits TPO-inducible megakaryocyte differentiation from CD34+ cord blood cells in vitro (Basu et al. 2008; Murgue et al. 1997; Nakao et al. 1989). Some reports also indicate increased clearance of circulating platelets either by platelet consumption due to coagulopathy or via activation of the complement system or by formation of anti-platelet antibodies by molecular mimicry (Alonzo et al. 2012; Honda et al. 2009; Lin et al. 2001; Lin et al. 2011; Srichaikul et al. 1989). It is probable that DENV-associated thromobocytopenia occurs due to both decreased production of cells from bone marrow and an increased peripheral destruction of platelets. Further research is warranted to assess if ZIKV uses mechanisms similar to DENV to induce thrombocytopenia.

In addition, there are multiple reports of ocular complications such as unilateral acute maculopathy, pigmentory retinopathy, and atrophy in infants with ZIKV infection (Parke et al. 2016). Macular and chorioretinal disease can significantly impact visual development in infant (Moshfeghi et al. 2016; Valentine et al. 2016; Ventura et al. 2016). Lastly, reports suggest an association between ZIKV infection and hearing loss. Leal et al. reported that prevalence of ZIKV-associated sensorineural hearing loss was 5.8%, similar to hearing loss associated with other congenital viral infections (Leal et al. 2016). Interestingly, Vinhaes et al. identified three cases of transient hearing loss in ZIKV-infected (Vinhaes et al, 2016). However, it is not yet clear if these complications are directly caused by ZIKV or are secondary to microcephaly. Nonetheless, in addition to brain malformations, all of these probable ZIKV-related health complications pose a serious concern and will affect the prognosis for ZIKV infection.

## Immune response against ZIKV infection

While there is a definite association between ZIKV infection and microcephaly, not all infants born to infected mothers have these complications, indicating that robust maternal immune responses might be able to protect the fetus from detrimental effects of ZIKV infection in some cases. Insights into ZIKV pathogenesis and immune correlates of protection can be gleaned from the prevailing mouse models as well as lessons learned from studies with other FVs. In vitro infection of fibroblasts causes an increase of antiviral pattern recognition receptors, such as TLR3 at an early stage and later RIG-I and MDA-5, leading to an induction of interferon alpha and interferon beta (Hamel et al. 2015). Mouse models have also been utilized to show the important immune cell types in ZIKV defense. Immunocompetent C57/BL6 and CD1 mice show no illness upon infection with ZIKV, whereas A129 mice and AG129 (IFN type I and II receptor knock out) mice developed signs of illness like hunched posture and ruffled fur, and the fetuses from infected mothers showed microcephaly (Rossi et al. 2016). It is worth noting that the mortality due to ZIKV infection in these experimental groups was not uniform, as few A129 mice with ZIKV died within 6 days, while other A129 infected mice survived the infection. Similar findings have also been reported with respect to DENV infection, where the innate immune response, especially type I and type II IFNs are postulated to orchestrate the disease outcome (Clyde et al. 2006; Rodenhuis-Zybert et al. 2010). Inoculation with the yellow fever YF-17D vaccine can delay the onset of dengue fever upon subsequent infection with DENV within a short window of vaccination (Liang et al. 2016a), further emphasizing the crucial role of innate immune response in defense against ZIKV.

SJL mice have also been used as a model for ZIKV infection. These mice exhibit normal IFN responses however show delayed B cell responses and lack natural killer cells (Hutchings et al. 1986; Sellers et al. 2012). Even though the fetuses from ZIKV-infected SJL dams did not develop microcephaly, they had cortical malformations, indicative of microcephaly (Cugola et al. 2016). This data points towards the requirement of NK cell-mediated immunity and neutralizing antibody response to combat ZIKV infection. A study with YF vaccine, YF17D, further confirms the importance of NK cell-mediated immune responses in anti-FV immunity wherein the authors showed that IFN-γ + NK cells and IL-4 + NK cells were significantly increased along with multiple NK-associated genes after the administration of this vaccine to healthy individuals (Gaucher et al. 2008; Silva et al. 2011). Another recent paper showed that T lymphocytes were not as important as neutralizing antibody response in ZIKV immunity (Larocca et al. 2016). In this study, the investigators produced a DNA vaccine expressing full-length ZIKV pre-membrane and envelope proteins and reported that a single injection with this vaccine provided complete protection against ZIKV infection in Balb/C and SJL mice upon viral challenge. Adoptive transfer of purified IgG from immunized mice conferred passive protection, while depletion of CD4 and CD8 T lymphocytes in vaccinated mice did not affect protective efficacy of the vaccine, indicating that ZIKV envelope-specific antibody response might be the correlate of immune protection. This finding was further supported by one more report, in which the researchers developed a panel of anti-ZIKV monoclonal antibodies by inoculating irf^−/−^ mice with live virus followed by boost with either the virus or E proteins. They found that the antibodies against the DIII domain of E protein not only possessed neutralizing activity but also protected IFN-deficient mice from lethal infection (Zhao et al. 2016). Overall, these studies highlight the role for innate and antibody-mediated immune responses in anti-ZIKV immunity, and a candidate vaccine that will stimulate these types of immune responses might be highly desirable.

## Current anti-ZIKV vaccine efforts and comparison with other FV vaccines

The use of vaccines to prevent viral infections is the most cost-effective public healthy strategy, and vaccines are currently in use for other FVs, including YF, JEV, and tick-borne encephalitis viruses (TBEV) (Ishikawa et al. 2014). The YF-17D vaccine for yellow fever is one of the most successful anti-FV vaccines and an excellent roadmap for generalization of immune correlates of protection against FVs (Pulendran 2009; Pulendran et al. 2013). The rubella pandemic from 1962 to 1965 caused congenital rubella syndrome, including fetal impairments like deafness and developmental delays, in 20,000 infants in the USA. Since the development and implementation of the rubella vaccine, these numbers have fallen to less than ten cases per year (Martinez-Palomo 2016). Preliminary work using a guinea pig model for CMV, which causes mental retardation and deafness in infants, showed that vaccination was able to reduce transmission and mortality in pups caused by CMV infection (Choi et al. 2016). In 2015, the first vaccine for prevention of DENV, Dengvaxia, was licensed for persons aged 9–45 years living in DENV endemic regions. It is a chimeric yellow fever-DENV tetravalent live-attenuated vaccine and is estimated to reduce disease burden by 10–30% over a period of 30 years. It is also associated with high risk of hospitalization for children under 9 years of age. Further, Dengvaxia seems more effective against secondary DENV infection and has lower efficacy when given to dengue-naïve people (WHO 2016b). A successful vaccine candidate might be expected to provide similar relief from negative congenital effects associated with ZIKV infection during pregnancy.

Current anti-ZIKV vaccine strategies utilize diverse range of molecular approaches. In March 2016, the World Health Organization compiled a list of 18 active programs (including 5 academic and 15 commercial groups) pursuing different strategies in parallel. Approaches used include live-attenuated virus, nucleic acid based, live vectors, subunit vaccines, and nanoparticles (WHO 2016a). In June 2016, Inovio Pharmaceuticals announced that they have received approval to initiate phase I human trial to evaluate the DNA vaccine (GLS-5700) produced by Larocca et al. (Larocca et al. 2016; Morrison 2016). Further, Sapparapu et al. isolated broadly neutralizing anti-ZIKV antibodies from infected individuals and showed that preimmunization with these antibodies, significantly reduced placental transmission of ZIKV in mouse models (Sapparapu et al. 2016). However, while animal and small sample trials for these and other candidate vaccines might be completed very soon, long-term efficacy trials and approval are likely to take years. At the same time, the possibility of causing antibody-dependent enhancement (ADE) of DENV infection by ZIKV vaccine and vice versa will also impact the development of a successful vaccine.

## Antibody-dependent enhancement of infection: potential roadblock in development of ZIKV prophylaxis

The recently licensed DENV vaccine is partially successful and can be used only in a certain population (aged 9–45 years) living in highly endemic areas (WHO 2016b). Unlike YF and JEV vaccines, the development of a successful DENV vaccine has proven challenging due to the existence of four divergent serotypes of DENV. Epidemiological evidence suggests that primary infection confers protection against reinfection with the same serotype but can cause severe disease upon reinfection with a different serotype, owing to ADE. ADE occurs when antibodies generated during primary infection fail to neutralize the virus of a different serotype during secondary infection, instead causing enhanced infectivity of target cells through virus opsonization and Fc-receptor-mediated endocytosis (Halstead 2014). Phylogenetic analyses of human pathogenic FVs using RNA polymerase NS5 indicate that ZIKV clusters with the encephalitic viruses; however, when the E protein sequence is considered, it branches with DENV group (Barba-Spaeth et al. 2016). The four DENV serotypes differ from each other by 30–35%, and this serocomplex in turn is different from ZIKV by 41–46% (Dejnirattisai et al. 2016; Screaton et al. 2015). This difference indicates that vaccine design against ZIKV infection might not be as straight forward as with YF or JEV. Multiple recent reports confirm that this divergence in structure is not just a theoretical obstacle but is causing actual issues in vaccine development.

Barba-Spaeth and coworkers identified structural details of a quaternary epitope in DENV known as envelope dimer epitope (EDE) formed at the interface of two enveloped monomers, 90 of which are arranged in icosahedral symmetry into the DENV glycoprotein shell. Antibodies targeting this region can be divided into two categories: EDE1, which is not sensitive to glycosylation at the Asn153 residue and EDE2, which requires the N-linked glycan at Asn153. They found that antibodies targeting EDE1, especially C8 and C10, efficiently neutralized ZIKV, while other antibodies targeting the fusion loop epitope were just cross-reactive (Barba-Spaeth et al. 2016). A study by Dejnirattisai et al. showed that multiple anti-DENV antibodies not only fail to neutralize the infection but also cause ADE (Dejnirattisai et al. 2016). In this study, preincubation of ZIKV with anti-dengue immune sera or well-characterized anti-dengue monoclonal antibodies (including EDE1 C8 and C10) lead to increased infection of U937 monocytic cells with ZIKV. However, for unknown reasons, the combination of both these immune components was able to neutralize ZIKV.

Swanstrom et al. confirmed the neutralizing potential of these two antibodies in human monocytic cell line, U937-DC-SIGN, and Vero cells (Swanstrom et al. 2016). In addition, IFN-type I/II receptor knockout mice, when pretreated with C10 antibody, exhibited no signs of illness upon infection with ZIKV. They also found that antibodies, which are capable of neutralizing only one or two DENV serotypes, failed to neutralize ZIKV. Further, in the same study, sera obtained from DENV-infected individuals (with one or more than one serotypes) several years postinfection, failed to cross-neutralize ZIKV. Similar results were noted by Priyamvada and her team in which they tested acute and *convalescent* sera from patients with DENV infection and showed that while some anti-DENV antibodies confer neutralization of ZIKV infection, most antibodies caused ADE (Priyamvada et al. 2016). In yet another study by Stettler et al., 119 anti-ZIKV monoclonal antibodies were isolated from two ZIKV-infected, DENV-naïve individuals and two ZIKV-infected, DENV-immune donors and compared with a panel of mAbs previously isolated from ZIKV-naïve, DENV-infected donors. Of the 119 antibodies, 41 were against NS1 and the rest were against E protein. Of the NS1 antibodies, only the ones from ZIKV-infected, DENV-immune individuals were cross-reactive, while antibodies from ZIKV-infected, DENV-naïve, and DENV-infected, ZIKV-naïve individuals were reactive only to antigens from specific viruses. Interestingly, the anti-E antibodies from ZIKV-infected, DENV-naïve individuals cross-reacted with E protein of the four DENV serotypes and vice versa. They also found that cross-reactive antibodies elicited by either viruses and primarily directed to EDI/II domains could mediate heterologous ADE, while antibodies against EDIII and quaternary epitopes present on infectious virus cause potent neutralization (Stettler et al. 2016). While contradictory in some aspects, all of these reports elucidate that antibodies against different serotypes of DENV and ZIKV are definitely cross-reactive. These studies reveal the complexity of a potential antibody-based vaccine for ZIKV, especially as both of these viruses are prevalent in the same geographical areas. This also means that it might be necessary to explore the potential of therapeutic drugs for treatment of ZIKV infection.

## Pharmacological interventions against ZIKV infection

Prevention of mother to fetus transmission via the use of an entry inhibitor would be the ideal solution to avoid cortical complications in the infants. Recent reports suggest that Axl is the primary entry receptor used by ZIKV in neuronal as well as skin cells (Hamel et al. 2015; Nowakowski et al. 2016), while placental cells exhibit consistently high levels of TIM1 receptor and that is utilized by ZIKV to infect these cells (Tabata et al. 2016). Similar results were also obtained by Sapparapu et al. via injection of broadly neutralizing anti-ZIKV antibodies from infected humans into pregnant mice (Sapparapu et al. 2016). Further, Tabata et al. were able to significantly reduce the ZIKV infection of early and mid-gestation placental explants by using Duramycin, an inhibitor of TIM1 receptor, while the Axl inhibitor R428 had a modest effect. Duramycin is also known to inhibit infection by DENV, WNV, and Ebola virus (Richard et al. 2015), indicating that use of entry inhibitors might prove be an effective strategy to prevent transplacental transmission of ZIKV. Using a different approach, Barrows et al. screened a library of FDA-approved drugs for their ability to block in vitro infection of HuH-7 cell line by ZIKV and found 20 plausible candidates (Barrows et al. 2016). Some of these candidates were further validated for inhibition of ZIKV infection in human cervical, neural, and placental cells. The candidate compounds include few previously known anti-flaviviral drugs like bortezomib, while some were newly identified to have antiviral activity like daptomycin and sertraline. On similar lines, Xu et al. designed a compound screening approach using caspase-3 activity as the primary screening assay and confirmed the neuroprotective nature of the identified compounds by a secondary cell viability assay. This lead to identification of two types of drugs, antiviral, and neuroprotective. Emricasan, a pan-caspase inhibitor, was identified as the most efficient prosurvival compound, while niclosamide and PHA-690509 were found to have potent antiviral activity. Interestingly, a combination of the two types of drugs helped the infected neural progenitor cells recover by preventing their apoptosis (Xu et al. 2016).

Repurposing of FDA-approved drugs to identify anti-ZIKV compounds is an advantageous strategy that has proven successful in case of other infections such as Ebola and hepatitis C virus (He et al. 2015; Johansen et al. 2015) and especially required in case of ZIKV infection because there are no approved anti-flavivirus treatments and development of an efficacious vaccine will take a long time. Combining antiviral compounds with a neuroprotective drug might also prove extremely beneficial, but with a caution, that use of a pan-caspase inhibitor might have detrimental side effects on brain development. Induction of apoptosis is necessary during neurodevelopment to prevent brain overgrowth as well as establishment of appropriate neuronal connectivity and synapse formation (Yamaguchi and Miura 2015). Instead, pharmacological agents that activate signaling pathways involved in neurodevelopment and survival of neuronal progenitors, such as sonic hedgehog (Shh) signaling, might prove an interesting avenue of pursuit. This signaling pathway is crucial for maintenance of neural progenitor pool in fetal and adult brains (Machold et al. 2003). Further, our group has shown the neuroprotective effects of Shh pathway augmentation via a small molecule, smoothened agonist (SAG), against human immunodeficiency virus (HIV)-associated neuropathology in humanized mice (Singh et al. 2016). Not only that, but SAG was also shown to prevent neuropathology in a rat model of spinal cord injury as well as a mouse model for Down’s syndrome(Bambakidis et al. 2010; Bragina et al. 2010), indicating that this approach might lead to timely identification of a neuroprotective drug in order to dampen ZIKV-associated neurological complications.

## Concluding remarks

ZIKV infection is spreading across the globe at an alarming pace not only in vector-prevalent areas but also to other countries like USA, because of a wider range of routes of transmission. What started as yet another tropical disease has ended up being a much more serious threat because of its association with neurologic complications. Based on our knowledge so far, an ideal ZIKV prophylactic or therapeutic agent will be required to satisfy various criteria such as,

1. Prevent ADE for ZIKV infection in DENV-immune individuals and vice versa.
2. Not increase the chances of developing/worsening GBS, which means that the use of live-attenuated vaccines may be ruled out until the cause of GBS is identified.
3. Safe for use in pregnant population and immune-compromised individuals especially because persons with immunosuppression or autoimmune disorders are at a higher risk of developing severe disease (Azevedo et al. 2016).
4. Keep in consideration the broader tropism as well as increased stability of the ZIKV and its effect on the different modes of transmission.

While development of a preventive vaccine candidate is extremely important, it is also necessary to focus attention on identifying pharmacological agents that can either prevent/reduce mother to infant transmission or confer neuroprotection in the presence of viral burden. Considering these multiple factors, it seems likely that a combination of these two approaches will be the most successful in eradicating this global threat.
